# Comparison of the Quality of Life and Depression in the Elderly with and without a History of COVID-19 Infection in Shiraz, Iran

**DOI:** 10.1155/2023/9991390

**Published:** 2023-03-07

**Authors:** Akram Boustani, Camellia Torabizadeh, Majid Najafi Kalyani

**Affiliations:** ^1^School of Nursing and Midwifery, Shiraz University of Medical Sciences, Shiraz, Iran; ^2^Community Based Psychiatric Care Research Center, Department of Medical Surgical Nursing, School of Nursing and Midwifery, Shiraz University of Medical Sciences, Shiraz, Iran; ^3^Department of Nursing, School of Nursing and Midwifery, Shiraz University of Medical Sciences, Shiraz, Iran

## Abstract

**Background:**

COVID-19 causes many physical and mental complications. The elderly, as one of the vulnerable groups, were more exposed to the problems caused by this pandemic. The aim of this study was to compare the quality of life and depression in the elderly with and without a history of COVID-19 infection.

**Method:**

This is a cross-sectional descriptive study conducted on 404 elderly people (202 from the affected group and 202 from the nonaffected group) aged over 60 years old in Shiraz city. The elderly participants were selected based on simple random sampling from the elderly list. In order to collect information, the quality of life questionnaire of the World Health Organization and Beck's depression questionnaire were used. Data analysis was done through SPSS software version 22 using statistical tests of chi-square, *t*-test, analysis of variance, and Pearson's correlation coefficient. An alpha level under 0.05 was considered the significant level.

**Results:**

The average score of depression in the elderly with a history of COVID-19 (14.66 ± 13.17) was significantly higher than that of the elderly without a history of COVID-19 (9.71 ± 10.12) (*p* < 0.001). The average score of the quality of life in the elderly with a history of COVID-19 (80.15 ± 14.85) was significantly lower than that of the elderly without a history of COVID-19 (85.25 ± 14.09) (*p* < 0.001).

**Conclusion:**

Elderly people with a history of COVID-19 had more depression and lower quality of life compared to people without a history of COVID-19. It is suggested that planners and health policymakers should pay special attention to the use of effective psychological interventions in order to reduce the problems of the elderly.

## 1. Introduction

After starting in China for the first time, the COVID-19 disease spread rapidly around the world and caused a pandemic [1]. Most people with this disease recover without any special treatment. However, the elderly and those with underlying medical conditions become more severely ill and are more susceptible to complications from the disease [2]. Old age is an important risk factor for severe infection with COVID-19, which causes other problems for the elderly, in addition to causing severe disease [3, 4].

This pandemic has had a lot of effects on all age groups in physical and mental dimensions [5–7], and the elderly have suffered the most [8]. Elderly people experience the most challenges and threats caused by this disease due to their susceptibility to more severe diseases and more deaths, as well as the consequences of social quarantine [9]. The fear and worry of catching the COVID-19 disease have a negative effect on the physical and mental health of the elderly [10]. One of the most common psychological problems in the elderly is depression, which can occur in any period of life, especially in critical situations [11]. A systematic review conducted by Bagheri Sheykhangafshe et al. showed that the spread of COVID-19 had a negative impact on various aspects of the mental health of the elderly [12].

Quality of life is one of the important concepts related to health, which is affected by time, place, and individual and social values [13]. In measuring health and evaluating health interventions, in addition to indicators of disease frequency and severity, other concepts related to health, such as quality of life, should also be considered [14]. The quality of life is affected by many factors, but psychological problems, including depression, have a great impact on it [11]. The elderly are at a greater risk of severe illness and death after contracting COVID-19, and this issue makes them prone to increasing psychological problems and reducing the quality of life [15]. Studies have shown that the quality of life of the elderly has decreased during the COVID-19 pandemic compared to before the pandemic [16, 17].

Given the importance of the impact of the COVID-19 pandemic as a situational-social crisis that has affected the lives of all people around the world and the greater vulnerability of the elderly, investigating and identifying health-related problems in this group are necessary for planning and future policies. A review of the conducted studies shows that although the influence of people in terms of psychosocial health has been investigated, a study that specifically compares the level of depression and quality of life and related factors in Iranian elderly people with and without a history of COVID-19 has not yet been conducted. Given the existing information gap, this study was conducted with the aim of comparing depression and quality of life in the elderly with and without a history of COVID-19.

## 2. Methods

### 2.1. Study Design

This is a cross-sectional analytical study that was conducted on 404 elderly people with and without a history of the COVID-19 disease in a center affiliated to Shiraz University of Medical Sciences from June to October 2022 in the southwest of Iran.

### 2.2. Sample Size

The sample size was calculated based on a previous study [18] and considering alpha *α* = 0.05, beta *β* = 0.20, power = 80%, and 95% confidence coefficient using PASS 11 software for the number of 404 people (202 in each group).

### 2.3. Participants

The samples were selected based on random sampling from the list of the elderly people who referred to the treatment center and based on the inclusion and exclusion criteria. The criteria for entering the study included the elderly in the age range of over 60 years, willingness to participate in the study, a history of contracting the COVID-19 disease, and lack of a history of contracting the COVID-19 disease. Exclusion criteria included the presence of depressive disorder before contracting the COVID-19 disease and a history of taking neuropsychiatric drugs.

### 2.4. Procedure

After approving the project and obtaining the code of ethics from the university research ethics committee, the researcher referred to the medical center affiliated to Shiraz University of Medical Sciences, which was dedicated to the patients with COVID-19. Then, the participating elderly were selected based on simple random sampling from the list of those referring to that center. After obtaining the participants' contact, we contacted the eligible people for participating in the study. For people who were enrolled in the study, questionnaires and an informed consent form that was designed online (porsline) were sent through the WhatsApp social network and they were asked to complete the questionnaires. In order to select the samples without a history of COVID infection, 19 researchers were referred to a center affiliated to Shiraz University of Medical Sciences and invited patients to participate in the study. The selection of these old people was also based on the list of the elderly who had referred and based on simple random sampling.

### 2.5. Data Collection

Data collection in this study was done using three questionnaires. The personal information questionnaire included demographic characteristics such as age, gender, marital status, literacy, occupation, history of smoking, and history of opium use.

*World Health Organization Quality of Life Questionnaire (WHOQOL-BREF)*: this scale was created in 1996 by a group of experts of the World Health Organization and by adjusting the items of the 100-question form of this questionnaire. This questionnaire has 4 subscales and a total score. These subscales are physical health, mental health, social relations, health of the surrounding environment, and a general score [19]. The short form of this questionnaire contains 26 questions, each having options from 1 to 5, and the total score of the questionnaire after summing the subscales is between 0 and 100. The validity and reliability of this questionnaire were investigated by Vahedi in Iran. In this study, the correlation of this questionnaire was obtained in all areas above 70 using Cronbach's alpha [20].

*Beck depression questionnaire*: this questionnaire was designed in 1961 by Beck et al. and has 21 items. Each question has four options that people will choose according to the conditions [21]. The severity of depression in this scale ranges from 1 to 63, which will be classified into four states according to the obtained score [22]. The validity and reliability of this scale have been examined and confirmed in various studies [23].

### 2.6. Ethical Considerations

After explaining the purpose and method of conducting the study and obtaining an informed written consent, the elderly volunteers entered the study. This research has been approved and registered by the Research Ethics Committee of Shiraz University of Medical Sciences with code IR.SUMS.NUMIMG.REC.1401.026. All the elderly were given sufficient assurance about the confidentiality of the information. In addition, it was mentioned that they can withdraw from the study at any time if they were not willing.

### 2.7. Statistical Analysis

The collected data were analyzed through SPSS version 22 software using independent *t*-tests, analysis of variance, chi-square, and Pearson's correlation coefficient. An alpha level under 0.05 was considered statistically significant.

## 3. Results

This study was conducted on 404 elderly people with and without the history of the COVID-19 disease. The average age of the participants was 68.86 ± 8.27 years. The demographic characteristics of the participants are presented in Table 1.

As shown in Table 1, there was no statistically significant difference between the two groups in terms of gender (*p* = 0.135), marital status (*p* = 0.168), occupation (*p* = 0.083), opium use (*p* = 0.224), smoking (*p* = 0.443), and literacy level (*p* = 0.051) (Table 1).

According to Table 2, the examination of the two groups in terms of mean age, depression score, and quality of life showed that there was no statistically significant difference between the two groups in terms of mean age (*p* = 0.67), while in terms of the mean score of depression (*p* = 0.000) and quality of life (*p* = 0.000), there was a statistically significant difference between the two groups (Table 2).

## 4. Discussion

The present study showed the average depression and quality of life in Iranian elderly with and without a history of COVID-19. This is the first study in Iran that compares depression and quality of life of the elderly during the COVID-19 pandemic.

The results obtained from the present study showed that the average depression in the two groups of elderly people with and without a history of COVID-19 infection was different from each other. Various studies show that social isolation after contracting COVID-19 is one of the important risk factors for depression in the elderly [24, 25]. The results of Miklitz et al.'s study showed that the prevalence of depressive symptoms in the elderly during the COVID pandemic was 50.8% [26]. In line with the results of the present study, the findings of the study by Khademi et al. showed that patients with COVID-19 suffered from psychological disorders such as depression, which is more common in patients with a history of hospitalization than those without a history of hospitalization [27].

The results of several studies indicated an increase in depression in those who suffered from COVID-19. The range of depression in patients with COVID-19 in different countries has been reported between 4.28 and 57.53% [28–31]. Zaninotto et al. showed that depression (from 12.5% to 28.5%) increased significantly during the pandemic compared to before it [11].

Having appropriate psychological support protects the elderly against depression [30]. Madyaningrum et al. showed that the chance of depression had an inverse relationship with life satisfaction, health status, and physical condition of the elderly before the pandemic period [32]. In their study, Hosseini Moghadam et al. revealed that the fear of contracting COVID-19 infection caused an increase in depression in the elderly [33]. The COVID-19 pandemic is one of the important threats related to health, especially in the mentioned vulnerable groups [25, 34, 35]. It has been found that patients with COVID-19 are prone to more psychological problems due to self-quarantine and staying more at home [36, 37]. The fear and worry of the infected people about the transmission of the disease to other people as well as family members cause them to be worried that this problem can be the reason for more psychological problems in infected people [38]. Enduring high pressure, guilt, and stigma increases the risk of depression [39]. Contrary to the results of the present study, the study by de Almondes et al. showed that there was no difference in depression between people with COVID and noninfected people [40]. One of the reasons for this difference can be attributed to the small number of infected people (2.1%) compared to noninfected people (97.9%) in that study. Pascut et al. also showed that the physical and mental health status of the elderly had not been seriously disturbed since the start of the COVID-19 pandemic [41] since the aforementioned study was conducted in the third wave of COVID in Italy, but the present study was conducted in the seventh wave of COVID in Iran; this difference can be attributed to this issue. Given the disease and the mental and physical complications caused by it [42], the higher level of depression in the group with COVID-19 is justified.

Also, the results of the present study showed that the average quality of life in the two groups of elderly people with and without a history of COVID-19 infection is different from each other. The results of the study by Ma et al. revealed that patients suffering from COVID-19 with depression had a lower quality of life than patients without depression [43]. Since these patients suffer from physical problems due to the disease, the decrease in their quality of life can be justified [43]. In line with the results of the present study, the findings of the study by Ahi et al. indicated that the elderly with COVID-19 had a lower quality of life than the noninfected elderly. Also, there was a statistically significant difference between the score of the quality of life of the elderly before and during the COVID pandemic, so that the quality of life of the elderly during the pandemic was reported to be lower than before the pandemic [16].

The results of Ferreira et al.'s study showed that the quality of life of people quarantined due to the spread of COVID-19 was low [17]. Kharshiing et al.'s study showed that the fear of COVID-19 infection had a significant and inverse relationship with quality of life [44]. Zaninotto et al., in their study, showed that the quality of life of the elderly (decrease by 4.3%) during the pandemic significantly worsened compared to before the pandemic [11]. Given the disease and the mental and physical complications caused by it [42], the lower quality of life in the group affected by COVID-19 can be justified.

Higher depression and lower quality of life in elderly people with a history of COVID-19 infection in contrast with elderly without a history of COVID-19 infection are useful for health experts and health policymakers to make a comprehensive plan in the vulnerable groups.

## 5. Strengths and Limitations of the Study

The innovation and the most important strength of this study are the comparison of quality of life and depression in two groups of elderly with and without COVID-19, which was conducted for the first time in Iran. Since the data collection in this study was based on an online questionnaire sent to the elderly, the completion of the questionnaires by the elderly may have been accompanied by errors, which can be considered one of the possible limitations of this study.

## 6. Conclusion

In sum, given the higher mean score of depression and the lower mean score of quality of life in the elderly with a history of COVID-19 and the negative effects of these variables on the mental and physical health of the elderly, it is necessary to include the initial screening, long-term follow-up, and treatment of depression in the elderly with COVID-19 in the care plan of this vulnerable group in order to promote mental health and improve their quality of life. It is suggested that planners and health policymakers should pay special attention to the use of effective psychological interventions in order to reduce the problems of the elderly.

## Figures and Tables

**Table 1:** Demographic characteristics of the samples under investigation in two groups with and without a history of infection with COVID-19.

Group variable		History of COVID-19	Without history of COVID-19	*p* value
Gender	Female	106 (52.5%)	91 (45.0%)	0.135^∗^
	Male	96 (47.5%)	111 (55.0%)	

Marital status	Single	14 (6.9%)	20 (9.9%)	0.168^∗∗^
	Married	148 (73.3%)	156 (77.2%)	
	Divorced	4 (2%)	1 (0.5%)	
	Widow	36 (17.8%)	25 (12.4%)	

Level of education	Illiterate	60 (29.7%)	53 (26.2%)	0.051^∗∗^
	Elementary	53 (26.2%)	39 (19.3%)	
	Guidance	26 (12.9%)	24 (11.9%)	
	Diploma	31 (15.3%)	29 (14.4%)	
	Associate degree	16 (7.9%)	21 (10.4%)	
	Bachelor	16 (7.9%)	36 (17.8%)	

Job	Freelance	56 (27.7%)	59 (29.2%)	0.083^∗∗^
	Housewife	97 (48.0%)	74 (36.6%)	
	Employee	41 (23.3%)	62 (30.7%)	
	Military	6 (3.0%)	7 (3.5%)	

Opium use	No	186 (92.1%)	192 (95.0%)	0.224^∗^
	Yes	16 (7.9%)	10 (5.0%)	

Smoking	No	140 (69.3%)	147 (72.8%)	0.443^∗^
	Yes	62 (30.7%)	55 (27.2%)	

^∗^Chi-square; ^∗∗^ANOVA.

**Table 2:** Comparison of the two groups in terms of mean age, depression score, and quality of life.

Group variable	History of COVID-19	Without history of COVID-19	Mean difference	CI 95%	*p* value^∗^
	Mean ± SD	Mean ± SD			
Age	69.04 ± 7.78	68.69 ± 8.74	-0.35	-1.25, -1.97	0.57
Depression	14.66 ± 13.17	9.71 ± 10.2	-04.95	-2.65, -7.26	0.001
Quality of life	80.15 ± 14.85	85.25 ± 14.09	5.094	2.26, 7.92	0.001

^∗^ *T*-test.

## Data Availability

The data used for this research analysis are available from the corresponding author upon request.
